# Annotations

**Published:** 1910-04-30

**Authors:** 


### ANNOTATIONS.

### Pharmacists and Poison Labels.

An important decision has been given by the High Court regarding the labelling of poisons. A pharmacist carrying on business under a trading name, which was not his registered name, was summoned by the Pharmaceutical Society for selling a poison which was not labelled with his personal name, on the ground that this was an infringement of Section xvii. of the Pharmacy Act, 1868, which provides that all poisons shall be labelled with the name of "the seller." The magistrate held that the name of the seller was the personal name, and convicted. The defendant appealed to the High Court, which reversed this decision and decided that if a person is carrying on business under a trade name, it is compliance with the provision of Section xvii. if poisons are labelled with the trade name.

### Lactic Cheese.

The newspapers (at any rate the *Times*) are now publishing a full-page advertisement, addressed particularly to the medical profession, of a certain cheese, the consumption of which is claimed as fulfilling all requirements of the now popular "sour-milk" treatment. A good many testimonials from doctors appear, but it is stated that in deference to the strict etiquette governing the profession the names of these gentlemen are withheld. The name of one, however, is given, and that pretty prominently. His small book on the subject is styled a standard work by a well-known authority. We are not aware that this practitioner has had special experience in bacteriology and biochemistry, and, without charging Dr. Reinhardt with a desire to partake the gale of the discoveries of Metchnikoff, we might point out to him the rather false position in which he is placed by this firm's advertisement. It is a reflection on the critical faculty of the laity, but none the less a fact, that the production of any sort of brochure on a medical subject is enough to get a medical man great reputation among non-professional people.

### Grants to Medical Schools.

In reply to a question asked by Sir William Collins, the President of the Board of Education has stated that applications have been received by the Board of Education from the following medical schools for grants under the Regulations for Technical Schools, etc., in respect of the academical year 1909-10: In London—Charing Cross Hospital Medical School, London Hospital Medical School, London (Royal Free Hospital) School of Medicine for Women, St. George's Hospital Medical School, St. Mary's Hospital Medical School, St. Thomas's Hospital Medical School, University College Hospital Medical School, and Westminster Hospital Medical School; in the provinces—Bristol University Medical School, Durham University College of Medicine, Leeds University Medical School, Liverpool University Medical School, Manchester University Medical School, and Sheffield University Medical School. In the case of St. Mary's Hospital Medical School recognition was afforded in respect of the year 1908-9. In the case of all of them grants will become payable after the close of the present academical year—*i.e.* July 31, 1910—provided that the terms of the regulations are found to have been complied with in each case. The amount of grant which will be made in each case will be calculated in accordance with the terms of the regulations for technical schools, etc., having regard to the work done, and therefore cannot be stated until after the close of the academical year.

### The Health of the Nations.

Under this title the International Council of Women has issued, at the price of 1s., a book of reports received from the various affiliated National Councils in their respective countries setting forth the answers to certain inquiries which had been sent out to them from England. The fact that these inquiries were set on foot by the Countess of Aberdeen, the President of the International Council, is enough to assure their practical and useful character; it would indeed be difficult to name four more pressing problems in public health than those connected with the care of infants and children, the conditions under which women carry out industrial work, the housing of the people, and the prevention of tuberculosis. The answers received are on the whole encouraging; almost unanimously the reporters have improvement to record, though most of them have to admit the necessity for more of it. In respect to the conditions of women's employment there seems to be least amelioration; and if some countries have more progress to dwell upon than others, it is satisfactory to see that our own Colonies are well to the front in this matter. In fact, throughout the volume are to be found reports so interesting as amply to have repaid the trouble which the editor, Dr. Maria Ogilvie Gordon, has taken over their collection. The report on infant feeding in Italy is possibly the most amazing of all; it was compiled by Mme. Koenen, who sent out 100,000 circulars to doctors and midwives, and received 600 answers only. It is easy to understand, after reading this description, how it is that gastro-intestinal diseases claim such heavy toll, and indeed the principal wonder is that any children can be reared at all under the conditions described. It is possible that the author is unduly pessimistic, and shows the dark side of the picture only; for in America the experience of Drs. Cabot and Ritchie, summarised lately in these columns (The Hospital, April 9, 1910, p. 33), is much less unfavourable to the Italians and their methods of infant feeding. We can recommend this publication very strongly; it is evidence of really good work in a sphere wherein women are rightly prominent. The International Council is well advised in shelving the political and suffrage agitations which five years ago wasted so much of the time and energy that are now being bestowed on more practical and less controversial questions.

